# Postischemic Trigeminal Neuropathy Treated With Radiofrequency Ablation

**DOI:** 10.7759/cureus.37711

**Published:** 2023-04-17

**Authors:** Jonathan P Scoville, Nam K Yoon, Evan Joyce, Yair M Gozal, Philipp Taussky

**Affiliations:** 1 Neurosurgery, University of Utah, Salt Lake City, USA; 2 Neurosurgery, Beth Israel Deaconess Medical Center, Harvard Medical School, Boston, USA

**Keywords:** trigeminal rhizotomy, trigeminal neuropathy, trigeminal neuralgia, radiofrequency ablation, stroke, ischemia, glycerol rhizotomy, balloon rhizotomy

## Abstract

Trigeminal neuralgia is a pain syndrome that is defined by sharp electrical shock-like pain that radiates in the sensory distribution of the trigeminal nerve. The classical cause of this syndrome is vascular compression, but other causes, such as stroke, have also been described. Instances of post-ischemic trigeminal pain have been described as meeting the classic description, and are termed trigeminal neuropathy. The treatment paradigms for trigeminal neuralgia versus neuropathy differ significantly, especially with the consideration of surgical management.We present a case of a 78-year-old man with post-ischemic trigeminal neuropathy that was successfully treated with radiofrequency ablation after failure of conservative management.We also summarize three previous cases of post-ischemic trigeminal neuropathy that were also successfully treated with percutaneous surgical treatment, showing that percutaneous surgical management should be considered in patients with post-ischemic trigeminal neuropathy that fail conservative management.

## Introduction

Trigeminal neuralgia is characterized by paroxysmal sharp electrical shock-like pain that radiates in any combination of the trigeminal sensory distribution [1]. It is classically triggered by very light touch or by repetitive manipulations of the affected sensory distribution, such as tooth brushing or chewing [1]. It is most commonly caused by compression of the trigeminal nerve root, which in up to 90% of cases is from a vascular cause, although compression from aneurysm or tumor has also been identified [2]. Such cases have been categorized as classical trigeminal neuralgia [1-3]. Other causes of trigeminal pain have also been identified, such as multiple sclerosis and ischemic infarct, as well as other disorders affecting the normal trajectory of the nerve [3-7]. These cases have been categorized as trigeminal neuropathy [7]. Cases of trigeminal neuropathy secondary to ischemia reported in the literature have features that distinguish them from classical neurovascular trigeminal neuralgia [1-4,7-10].

Most cases of trigeminal neuropathy respond very well to conservative measures, and most reported cases of post-ischemia trigeminal neuropathy corroborate resolution of pain within six to eight weeks of treatment [1-3,8,10]; however, a minority of cases are refractory to conservative management and have been treated with surgical intervention. We report here a case of a 78-year-old man with left-sided trigeminal neuropathy that failed conservative management and was successfully treated with percutaneous radiofrequency ablation of the trigeminal nerve. To the authors’ knowledge, this is only the fourth reported case of ischemic trigeminal neuralgia in which successful surgical intervention has been used and the first in which percutaneous radiofrequency ablation was used.

## Case presentation

**Figure 1:**
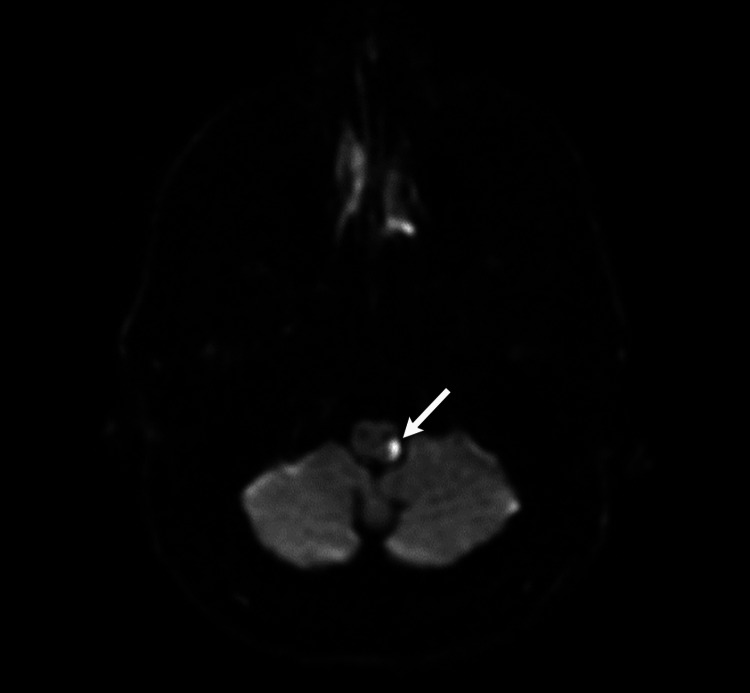
Diffusion-weighted MRI of the brain without contrast showing a small area of diffusion restriction in the left lateral medulla (arrow). Fast imaging employing steady-state acquisition and magnetic resonance angiography with contrast did not show any evidence of microvascular compression of the trigeminal nerve (not pictured).

The patient experienced a left lateral medulla stroke (Figure 1) and within the first 48 hours developed left-sided weakness, facial droop, and facial pain. This pain was described as lancinating and electrical, in the V2 and V3 distribution, and of 10/10 intensity; the pain was triggered by temperature changes and touching the left side of his face. The patient was diagnosed with post-ischemic trigeminal neuropathy. After trials and failures of treatment with various medications, including oxcarbazepine, baclofen, gabapentin, phenytoin, hydrocodone, tramadol, and amitriptyline, he was referred to neurosurgery for management of his trigeminal pain.

**Figure 2:**
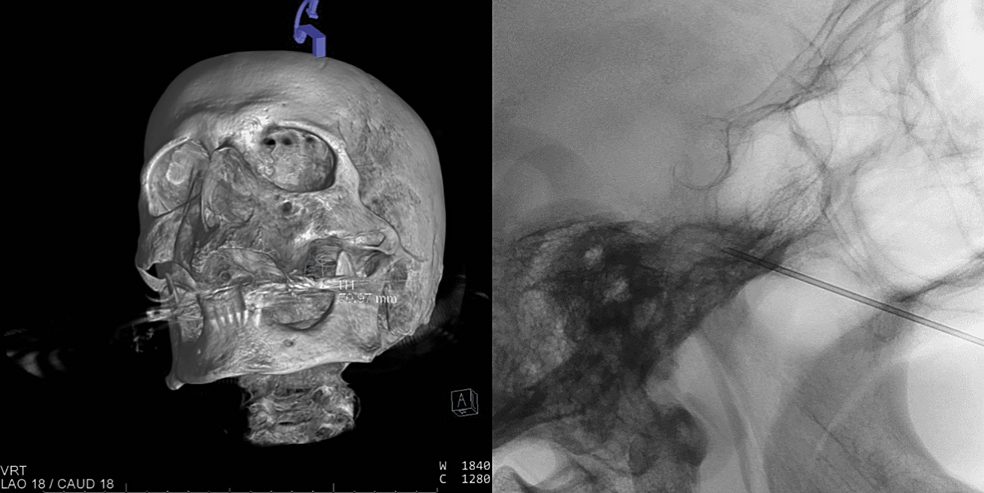
Imaging obtained during percutaneous glycerol neurolysis. (A) Rotational fluoroscopic spin with 3D reconstruction image showing the needle trajectory into the foramen ovale. (B) Lateral fluoroscopic image showing the 18-gauge spinal needle inserted through the foramen ovale in the trigeminal cistern.

Although the symptom onset and imaging findings were consistent with post-ischemic trigeminal neuropathy, the nature of the patient’s pain and aggravating factors were consistent with classic trigeminal neuralgia. After his treatment options were explained, because of the severity of his symptoms, the patient chose to proceed with percutaneous glycerol neurolysis of his trigeminal ganglion. This was performed in the interventional radiology suite, using a fluoroscopic spin with 3D reconstruction (Figure 2A). The patient was positioned in the sitting position. Needle guidance was used for stereotactic placement of an 18-gauge spinal needle into the trigeminal ganglion through the foramen ovale from a percutaneous entry site 1.5 cm lateral to the left side of his mouth (Figure 2B). Glycerol (0.4 mL) was injected into the trigeminal ganglion. The patient was kept in this upright position for one hour after the procedure and discharged home the same day.

After the procedure, the patient reported significant relief of his symptoms in the V2 and V3 distribution, but he started to notice pain in the V1 distribution. Because of the specific location of his symptoms, he wished to proceed with radiofrequency ablation of his trigeminal ganglion. Using the same technique described above, an 18-gauge spinal needle was advanced to the target, and then a radiofrequency ablation catheter was advanced to the target and unsheathed. The catheter was slightly repositioned to stimulate various areas of the face at levels ranging from 0.1 to 1.2 V. Once the areas of the face that corresponded to his pain were stimulated, ablation was performed with heating to 60°C for 60 seconds.

At his three-month follow-up, the patient reported complete symptom relief with only minor numbness in the left side of the face. The institutional review board did not require us to obtain consent for publication because the patient details are anonymized.

## Discussion

Trigeminal neuropathy tends to affect the V1 sensory distribution more commonly than V2 or V3, whereas classical trigeminal neuralgia is reported to more commonly affect the V2 and V3 distributions [8,10] (Table 1). The timing of onset of ischemic trigeminal neuropathy is another distinguishing factor from trigeminal neuralgia in that it typically presents within two weeks of the inciting infarct rather than as a sudden onset without inciting factors [8,10]. Most reported cases of trigeminal neuropathy from ischemia are found at the trigeminal nerve root entry zone or the principal sensory nucleus located in the pons; however, infarct along any portion of the trigeminal nerve pathway including in the medulla or along the spinal trigeminal accessory pathway has been reported to cause symptoms consistent with trigeminal neuropathy [1-3,6-10].

**Table 1:** Classic vs post-ischemia trigeminal neuralgia AEDs: anti-epileptic drugs

Characteristic	Classic	Post-ischemia
Symptoms	Repetitive paroxysmal electric pain lasting a few seconds	Repetitive paroxysmal electric pain lasting a few seconds
Distribution	V2, V3	V1, V2, sometimes V3
Imaging	MRI showing compression of trigeminal nerve root entry zone	MRI showing ischemia along the trigeminal nerve sensory pathway most commonly at the nerve entry zone in the pons
Treatment	Conservative treatment with AEDs or surgical intervention with percutaneous rhizotomy vs. microvascular decompression	Conservative treatment with gabapentin
Presentation	Sudden onset, no inciting factors	Within one month of infarct

The exact mechanism by which ischemia of the trigeminal nerve sensory pathway results in symptoms of trigeminal neuralgia is unknown; the most accepted hypothesis is that irritation from the glial scar and inflammation secondary to the infarct forms an epileptogenic focus causing random depolarizations, which are perceived as pain [3]. Conservative measures such as treatment with antiepileptic medications like carbamazepine and gabapentin are typically successful in treating trigeminal neuropathy, but cases may be refractory to conservative management and require surgery for successful resolution (Table 2).

**Table 2:** Case reports describing successful conservative treatment of patients presenting with post-ischemia trigeminal neuralgia

Study	Site of infarct	Treatment	Time until symptom resolution (weeks)
Nakamura et al. 1996 [10]	Medullary infarct	Gabapentin	3
Warren et al. 2006 [2]	Infarct of the right primary sensory nucleus of cranial nerve V	Gabapentin	3
Katsuno et al. 2010 [8]	9 cases of pontine infarct	Gabapentin	6
Ordás et al. 2011 [3]	Lateral medullary infarct	Gabapentin	3
Huang et al. 2015 [1]	Left lateral medullary infarct	Gabapentin	3

This is the fourth case of post-ischemic trigeminal neuropathy that has been successfully treated with percutaneous intervention (Table 3) [6,7]. The patient was originally treated with glycerol injection, which successfully alleviated his V2 and V3 pain but apparently unmasked his previously undescribed V1 pain. The V1 pain was then successfully treated with radiofrequency ablation. Percutaneous glycerol injection and radiofrequency ablation are proven treatments for trigeminal neuralgia [2,4,5,8,10]. These treatment modalities are minimally invasive and have low complication and high success rates [5]. The use of percutaneous trigeminal nerve treatment for post-ischemic trigeminal neuropathy should be considered in those patients in whom medical therapy is not adequate. Our patient had a left lateral medullary infarct that resulted in trigeminal neuropathy. His symptoms were classic for trigeminal neuralgia, but he lacked any evidence of vascular compression of the trigeminal nerve at the root entry zone.

**Table 3:** Case reports describing surgical treatment of patients presenting with post-ischemia trigeminal neuralgia in whom conservative management failed

Study	Site of infarct	Failed medical treatment	Surgical treatment
Foroohar et al. 1997 [6]	2 cases of pontine infarct in the trigeminal nerve root entry zone	Carbamazepine, Gabapentin	Radiofrequency ablation
Golby et al. 1998 [7]	Left pontine infarct of the trigeminal nerve root entry zone	Carbamazepine	Glycerol rhizotomy
This case	Left lateral medulla	Numerous (including oxcarbazepine, baclofen, gabapentin, phenytoin, hydrocodone, tramadol, and amitriptyline)	Percutaneous glycerol neurolysis; radiofrequency ablation

Three cases of post-ischemic trigeminal neuropathy successfully treated with percutaneous intervention have been reported (Table 3). Foroohar et al. [6] described a 50-year-old woman who developed right trigeminal neuropathy after stroke to the right pons. Initially, she responded to carbamazepine, but after seven months she re-presented with severe symptoms and failed conservative management with multiple medications. She then underwent a right-sided radiofrequency ablation and has remained symptom-free at six-year follow-up. Foroohar et al. [6] also reported a 76-year-old man who had left-sided trigeminal neuralgia after experiencing a stroke to his left lateral pons. Conservative management with multiple medications failed, and he underwent right-sided radiofrequency ablation and is pain-free at three-year follow-up. Golby et al. [7] described a 71-year-old man who presented with left trigeminal neuralgia after ischemic event to his left lateral pons. After failed conservative management, he underwent a left glycerol rhizotomy and reported 95% improvement of his pain at 13 months.

## Conclusions

Noncompressive causes of trigeminal pain, such as ischemia, are characterized as trigeminal neuropathy. Trigeminal neuropathy more commonly affects the V1 sensory distribution rather than the V2 or V3 distributions. Most cases respond to treatment with antiepileptic medications, but some are refractory to conservative management and require surgical intervention.

The four cases summarized here, including our case, suggest that patients that have classic trigeminal symptoms and radiographic evidence of stroke within the anatomic region of the trigeminal pathways should first be treated conservatively with gabapentin or carbamazepine. If no lasting benefit is achieved, then glycerol rhizotomy or radiofrequency ablation should be considered as an appropriate treatment option.
